# Vision Transformers (ViT) for Blanket-Penetrating Sleep Posture Recognition Using a Triple Ultra-Wideband (UWB) Radar System

**DOI:** 10.3390/s23052475

**Published:** 2023-02-23

**Authors:** Derek Ka-Hei Lai, Zi-Han Yu, Tommy Yau-Nam Leung, Hyo-Jung Lim, Andy Yiu-Chau Tam, Bryan Pak-Hei So, Ye-Jiao Mao, Daphne Sze Ki Cheung, Duo Wai-Chi Wong, James Chung-Wai Cheung

**Affiliations:** 1 Department of Biomedical Engineering, Faculty of Engineering, The Hong Kong Polytechnic University, Hong Kong 999077, China; 2 School of Engineering Sciences, Huazhong University of Science and Technology, Wuhan 430074, China; 3 School of Nursing, The Hong Kong Polytechnic University, Hong Kong 999077, China; 4 Research Institute of Smart Ageing, The Hong Kong Polytechnic University, Hong Kong 999077, China

**Keywords:** ablation study, deep learning, feature extraction, sleep monitoring, obstructive sleep apnea

## Abstract

Sleep posture has a crucial impact on the incidence and severity of obstructive sleep apnea (OSA). Therefore, the surveillance and recognition of sleep postures could facilitate the assessment of OSA. The existing contact-based systems might interfere with sleeping, while camera-based systems introduce privacy concerns. Radar-based systems might overcome these challenges, especially when individuals are covered with blankets. The aim of this research is to develop a nonobstructive multiple ultra-wideband radar sleep posture recognition system based on machine learning models. We evaluated three single-radar configurations (top, side, and head), three dual-radar configurations (top + side, top + head, and side + head), and one tri-radar configuration (top + side + head), in addition to machine learning models, including CNN-based networks (ResNet50, DenseNet121, and EfficientNetV2) and vision transformer-based networks (traditional vision transformer and Swin Transformer V2). Thirty participants (*n* = 30) were invited to perform four recumbent postures (supine, left side-lying, right side-lying, and prone). Data from eighteen participants were randomly chosen for model training, another six participants’ data (*n* = 6) for model validation, and the remaining six participants’ data (*n* = 6) for model testing. The Swin Transformer with side and head radar configuration achieved the highest prediction accuracy (0.808). Future research may consider the application of the synthetic aperture radar technique.

## 1. Introduction

Obstructive sleep apnea (OSA) is one of the most common sleep breathing disorders, with a prevalence of 9% to 38% that increases with age [1]. Untreated OSA patients may stop breathing numerous times every night when they sleep [2]. In order to “restart” breathing, the brain awakes, which leads to poor and fragmented sleep [2]. Sleep apnea has serious health repercussions and elevates the risk of diabetes, heart disease, hypertension, and heart failure if left untreated [3]. The care of concomitant neurological diseases, such as epilepsy, stroke, multiple sclerosis, and headache also becomes burdensome [4]. OSA has caused an economic burden of USD 6.5 billion in accidents and injuries, USD 86.9 billion in lost productivity at work, and USD 30 billion in healthcare annually in the USA [5]. 

There is an established relationship between OSA and sleep positions/postures [6]. A supine posture might significantly reduce the risks of OSA because it prevents the prolapse of the tongue and the soft palate against the pharyngeal wall by gravity [6,7]. In addition, a prone position presses on the lungs and affects respiration [7]. Contrarily, a lateral position (or side-lying posture) resolves the issue by maintaining the retropalatal and retroglossal airways [8]. These findings also support the crucial impact of the sleep position on the incidence and severity of sleep apnea [6,7,8]. In order to assess the sleep positions of OSA patients and their rehabilitation progress, sleep posture recognition and tracking could be one of the essential assessment components [9].

Various sensors have been developed to monitor sleep postures and behaviors, including body pressure sensors, physiological sensors, cameras (and depth cameras), and wearable devices [10]. The pressure intensity distribution generated by a pressure mat has been utilized to characterize sleep postural behavior and estimated sleep quality [11,12,13]. Video recordings using red–green–blue (RGB) or red–green–blue–depth (RGB-D) images can capture and facilitate observation of the sleep postures of individuals directly [14,15,16,17]. Wearable devices using actigraphy or accelerometry can measure physical activity and infer motor or behavioral activities [18]. Therefore, spectrogram analysis of data from wearable devices can be used to estimate sleep postures via the movement of body segments [19]. However, these systems or sensors can be expensive or interfere with sleep, which discourages practical use. Optical sensors or cameras suffer from interference from ambient light sources [20], in addition to privacy concerns [21].

Radar-based techniques might overcome these challenges and have demonstrated applications to sleep posture recognition [22]. In fact, there are different kinds of radar signals. Continuous wave radar is the most common type that sends and receives frequency signals continually, but it has poor discriminability with pulse and respiratory signals [22]. Frequency-modulated continuous wave (FMCW) radar cannot remove the weakness and is vulnerable to radio incoherence from unsteady object motion and micro-Doppler signals [23]. Impulse radio ultra-wideband radar (IR-UWB) can detect multiple objects and evaluate distance with less radiation [23], which facilitates applications, such as in body movement analyzers and trackers [24,25]. Ahmed and Cho [26] analyzed the waveform of IR-UWB to differentiate hand movements and gestures. Rana et al. [27] developed a markerless gait analysis system using IR-UWB technology. Recently, Lai et al. [28] attempted to classify sleep postures using IR-UWB signals by identifying their key statistical features.

The posture recognition function is often facilitated by machine learning techniques, especially tree-based and convolutional neural network (CNN) models. Piriyajitakonkij et al. [29] designed a CNN model, SleepPoseNet, that applied a feature-mapped matrix of time and frequency domains to estimate transitional postures. Kiriazi et al. [30] applied the decision tree to the effective radar cross section (ERCS) and displacement magnitude information to distinguish stationary torso postures. Zhou et al. [31] transformed a radar signal to image features, which were then handled by a CNN model integrated with an inception-residual module. Tam et al. [14] guided a CNN-based deep learning model (ECA-Net) by generating anatomical landmarks on depth images using a pose estimator. In addition, using random forest, Lai et al. [28] extracted radar features from each radar bin range for posture recognition. Although CNN models are widely used because of their capability to understand higher level semantic features and their superior performance [32], they were poor in understanding the global representation that might affect the performance of sleep posture recognition. 

A branch of deep learning models, vision transformers (ViTs), has emerged recently [33,34,35]. The origin of ViT, “Transformer”, was designed for natural language processing (NLP) and was later applied to visual computing tasks, such as object detection [36] and segmentation [37], and human motion recognition [38], such as pose estimation [39,40], and gait recognition [41,42]. The “Transformer” built upon the sequence-to-sequence encoder–decoder architecture and substituted the recurrent layers with attention mechanism, enabling the long-term memory of every token (word) [43]. While CNN applied pixel arrays in the model and lost spatial relationship information in the pooling layers [44], ViT has a substantially different backbone and model architecture from CNN models. It embeds and segments images into small patches followed by a self-attention mechanism without the use of convolutional layers [34]. The patches and positional embedding are input to the transformer encoder, which originally operates on tokens (words) [34]. ViT has a higher computational efficacy and accuracy than CNN models but requires more training data [45,46]. 

**Figure 1:**
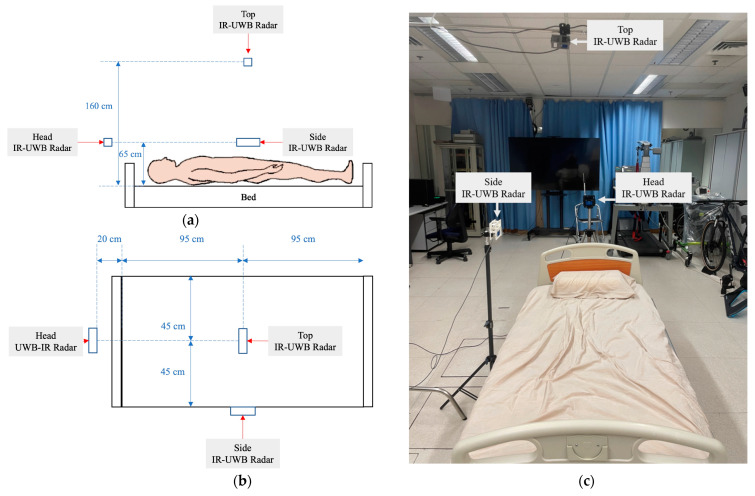
Schematic diagram of the system setup in (**a**) side view; (**b**) top view; and (**c**) photo.

Our study was motivated by the need for sleep posture assessment for OSA patients that might not be practically fulfilled by the current systems because of cost, privacy concerns, and the interference with sleep. The novelty of this study lies in the transformation of multiple radar signals to a spatiotemporal graph that can be input to the cutting-edge deep learning model, ViT, for sleep posture recognition. As we were interested in different configurations of the radar systems, the radars were placed on the ceiling (top radar), at the side of the participant (side radar), and on top of the head of the participants (head radar), as shown in Figure 1. We assumed that the tri-radar configuration (top + side + head) could improve the accuracy of posture prediction as compared to the single radar (top, side, and head) and the dual-radar configurations (top + side, top + head, and side + head). In addition, we compared different deep learning models, including CNN-based models (ResNet [47], DenseNet [48], and EfficientNet [49]) and ViT (traditional ViT [34] and Swin vision transformer [50]). We hypothesized that vision transfers with the tri-radar configuration (top + side + head) would outperform the others.

## 2. Materials and Methods

### 2.1. Hardware and Software Configuration

Three IR-UWB radar sensor system-on-chips (Xethru X4M03 v5, Novelda, Oslo, Norway) were used. Each sensor consisted of a fully programmable system controller and an antenna. The transmitter center frequency and energy per pulse were 7.29 GHz and 2.65 picojoules, respectively, which complied with the ETSI/FCC. The receiver had a sampling rate of 23.328 GS/s, a total radar frame length of 9.87 m, and the distance between each radar bin was 0.00643 m. The receiver gain noise figures were 14.1 dB and 6.7 dB, which also met the ETSI/FCC compliance requirement. Both the range of elevation angle and the azimuth angle were between −65° and +65°. The other parameters are shown in Table 1. The detection range was adjusted to encompass the region of interest (RoI).

### 2.2. System Setup

The system setup involved three IR-UWB radars with associated connection cables, a height adjustable hospital bed, a comforter, two tripods, one light boom, and a laptop computer. As shown in Figure 1, the bed was 0.5 m from the ground. The top radar was hung on a light boom, which was 1.5 m from the bed surface close to the height of a household ceiling. The side radar was mounted 0.65 m from the bed surface using a tripod, at the longer edge of the bed, according to the minimum detection distance requirement of the IR-UWB radar. The head radar was mounted 0.65 m from the bed surface and 0.2 m from the shorter edge using another tripod, to accommodate the effective angle of the depression (60°) of the radar. The three sensors were positioned orthogonally over the hospital bed (1.9 m × 0.9 m × 0.5 m with mat).

### 2.3. Participant Recruitment and Data Collection

All recruited participants signed an informed consent after receiving an oral and written description of the experiment before beginning the experiment, which was approved by the Institutional Review Board (reference number: HSEARS20210127007). The inclusion criteria included healthy adults aged over 18. In this study, we recruited 30 young adults (19 males and 11 females). Their average age was 22 (SD: 2.00, range 18–27). The mean weight and height were 170 cm (SD: 9.64 cm, range 156.5–196 cm) and 62.5 kg (SD: 12.70 kg, range 46–100 kg), respectively. The exclusion criteria included physical disability, obesity, pregnancy, or any cardiorespiratory problems, in addition to participants with difficulties in maintaining or switching specific postures in bed.

**Figure 2:**
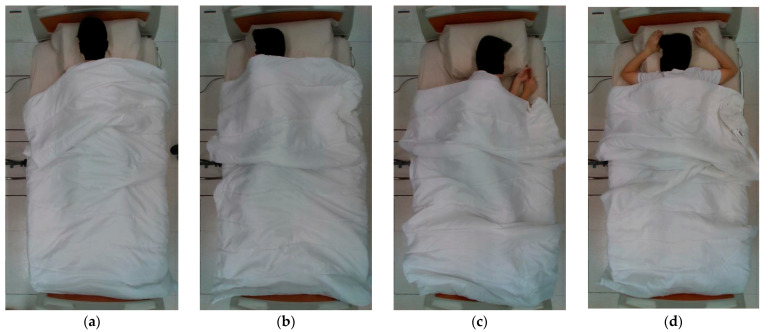
The four under-blanket recumbent postures: (**a**) supine; (**b**) right side-lying; (**c**) left side-lying; (**d**) prone.

Before the experiment, participants were instructed to remove clothing or accessories with metallic components (such as a belt with a metallic buckle), in addition to their shoes and outerwear. Throughout the experiment, they were asked to lie on the bed with a support pillow, covered by a comforter. They were then instructed to lie in different postures, in the order of (1) supine, (2) right lateral, (3) left lateral, and (4) prone, as shown in Figure 2. A ringing bell was played to notify the participants to adopt their assigned posture with self-chosen comfortable limb placement. After the participants finalized their posture, we then started the recording. Each posture was recorded for 15 s. The full course was repeated ten times. We collected 1200 samples (30 participants × 4 postures × 10 repetitions). The samples were labelled manually during the experiment.

### 2.4. Data Processing

The data processing pipeline comprised preprocessing, denoising, augmentation, resizing, and merging. The top and side radars produced 78 bins per frame, while the head radar produced 125 bin per frame. In the preprocessing stage, the last ten seconds of the recording were extracted for analysis. In the denoising stage, static objects in the scene were removed using the mean subtraction method by taking the average and subtracting it from each sample (Equation (1)). Then, background suppression was performed to remove the environmental noise (Equation (2)).

(1) $$X^{\prime }\left[n,m\right]=X\left[n,m\right]-\frac{1}{N}\overset{N-1}{\underset{i=0}{\sum }}X\left[n,i\right]$$

(2) $$Y\left[n,m\right]=X'\left[n,m\right]-\frac{1}{M}\overset{M-1}{\underset{i=0}{\sum }}X\left[i,m\right]$$

where *n* was the fast time index (radar bin), and *m* was the slow time index (frame number).

**Figure 3:**
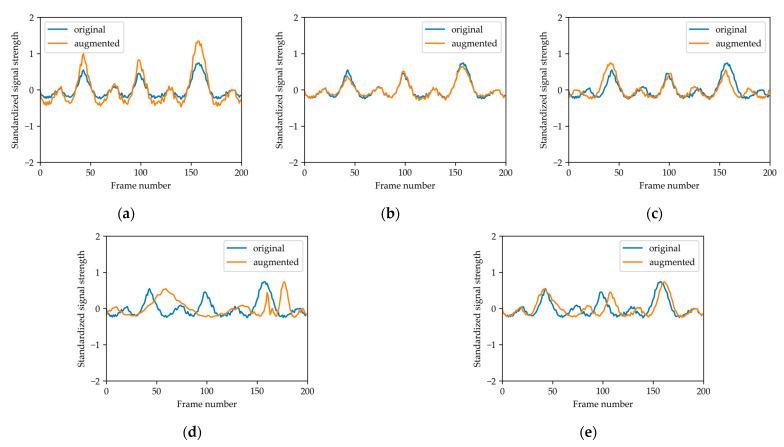
The five data augmentation strategies applied in our system, including: (**a**) scaling; (**b**) magnitude warping; (**c**) flipping; (**d**) time warping; and (**e**) window warping.

Data augmentation techniques, including scaling, magnitude warping, flipping, time warping, and window warping [51,52] were applied (Figure 3). The scaling process multiplied each frame of the signal by a random scalar. For magnitude warping, the time series of each bin was multiplied by a curve generated using a cubic spline of 4 knots and sigma = 0.2 [51,52]. The flipping process flipped at the center timepoint. Time warping perturbated the signal using the magnitude wrapping curve; while for window warping, a random window of 10% of the original duration wrapped the time dimension by 0.5 times or 2 times [51,52].

**Figure 4:**
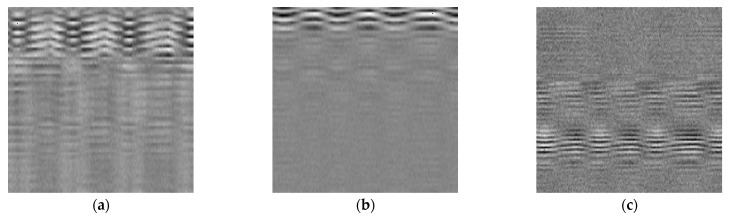

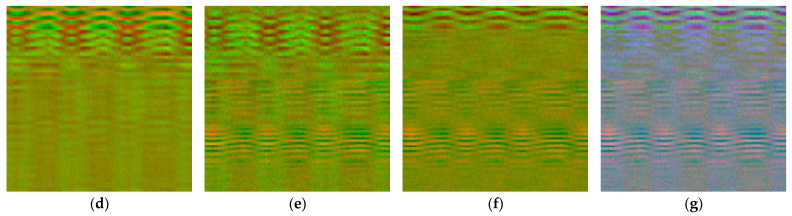
The imitated images generated by the single-radar configurations: (**a**) top radar, (**b**) side radar, and (**c**) head radar; the dual-radar configurations: (**d**) top + side radars, (**e**) top + head radars, (**f**) and side + head radars; and the tri-radar configurations: (**g**) top + side + head radars. The *x*-direction of the image represents the bin resolution, and the y-direction represents time. The resolution of the images was unified and resized to 224 pixels × 224 pixels based on the data of the radar.

The augmented data were resized to images of 224 pixels × 224 pixels to accommodate the input size requirements of the deep learning model (Figure 4). In the merging stage, the data of the three radars were distributed to three channels (RGB) to imitate an image. For the single-radar configuration, the data were cloned for the three channels. For the dual-radar configuration, the data of the first radar were input to the red channel, while those of second radar were input to the green channel, and a 224 × 224 zeroes array was assigned to the blue channel. For the tri-radar configuration, the top, side, and head radar corresponded to the red, green, and blue channels, respectively. Figure 4 illustrates the imitated image visualization of different radar configurations.

### 2.5. Model Training

The models were pretrained by ImageNet [53]. We trained the model using the data of 18 randomly selected participants, validated the model using the data of six participants, and tested it using the data of the other six participants. The performance from the three convolutional-based models (ResNet50, DenseNet121, and EfficientNetV2) and the two attention-based models (vision transformer and Swin Transformer V2) was compared in this study. The cross entropy loss was regarded as the loss function. The stochastic gradient descent using an initial learning rate of 0.001 and a momentum of 0.9 was applied as the optimizer. The learning rate was scaled down 10 times every 20 training epochs. Every model was trained for 100 iterations.

### 2.6. Model Validation

We used accuracy as the primary outcome of the models, which was defined by the fraction of correct predictions over the total number of predictions on the testing set. The validation dataset adjusted the model weights and attempted to minimize overfitting by facilitating early stopping. The accuracy was calculated by comparing the model prediction with the testing dataset.

## 3. Results

### 3.1. Performance of Different Models

**Figure 5:**
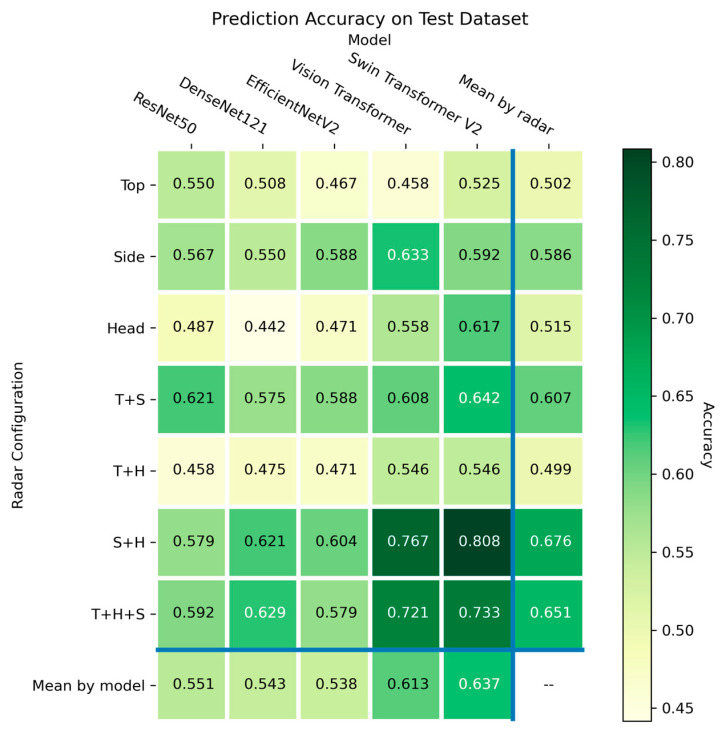
Heatmap showing the prediction accuracy of the different machine learning models and radar configurations. H: head radar; S: side radar; T: top radar.

The transformer-based models performed generally better than the convolutional-based model, where the average accuracies were 0.613 and 0.637 for the vision transformer and Swin Transformer, respectively, compared to 0.551, 0.543, and 0.538 for the ResNet50, DenseNet121, and EfficientNetV2, respectively, as shown in Figure 5. Among all models, the Swin Transformer with the side + head radar configuration produced the best prediction accuracy (0.808).

### 3.2. Performance of the Radar Configurations

Overall, the dual-radar was able to produce the better result, followed by the tri-radar and the single-radar. From Figure 5, the average accuracies of the dual-radar configurations were 0.676, 0.607, and 0.499 for the side + head, top + side, and top + head configurations, respectively, compared to 0.651 for the tri-radar configuration, and 0.586, 0.515, and 0.502 for the side, head, and top configuration, respectively. Among all configurations, the side + head configuration with Swin Transformer yielded the best accuracy (0.808).

### 3.3. Subgroup Analysis on Posture Conditions

**Figure 6:**
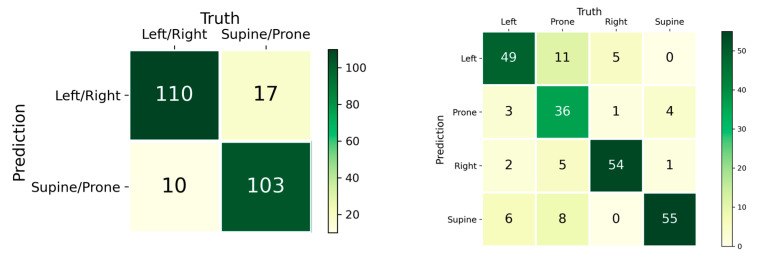
Subgroup analysis of the posture conditions using a confusion matrix over the prediction performance of the Swin Transformer with dual-radar configuration (side + head).

We extracted the confusion matrix of the Swin Transformer with the side + head radar configuration, which had the best prediction outcome (Figure 6). For a total of 120 predictions, the side-lying postures had 110 correct predictions, while the supine/prone had 103 predictions. The supine postures gave the largest number of correct predictions (55/60), followed by right side-lying (54/60), left side-lying (49/60), and prone (36/60).

## 4. Discussion

The novelty of this study lies in the application of the vision transformer deep learning models and multiple radar configurations to improve the sleep posture classification accuracy. The challenges of the radar system were that it could not effectively distinguish a stationary target from clutter, though existing radar processing techniques have mainly focused on moving target detection [54]. In addition, radar processing often relies on frequency analysis, such as fast Fourier transform (FFT), which could be more sensitive to dynamics and biomotions (respiration and heartbeat) but relatively insensitive to stationary postures [54]. We addressed these challenges by applying multiple radar systems that could reflect the different body cross-sectional areas in different postures.

Both the convolutional-based and transformer-based models were capable of extracting the pattern, but the transformers could further facilitate positional encoding [55]. In particular, this mechanism allowed the transformer models to locate the fringes, which was an important feature to distinguish different postures. Among the two transformer models, the Swin Transformer utilized shifted window and masked signal modeling techniques. On the other hand, the CNN models, such as EfficientNet, used the compound scaling method, by optimizing the network depth, network width, and input image resolution [56]. Nevertheless, the imitated images had low resolution, and the potential of EfficientNet could not be unleashed. In contrast, ResNet and DenseNet were designed for an input image with lower resolution; their higher performance over EfficientNet reflected that the two models might be more suitable in our image resolution setting. ResNet and DenseNet differ in how they connect the feature maps. ResNet sums all feature maps, while DenseNet concatenates them [57]. In our study, ResNet had superior performance on single radar settings, but DenseNet was better on dual-radar and tri-radar settings, showing that the summation approach might work better on single radar settings, and concatenation might work better on dual-radar and tri-radar settings.

Our results indicated that the configurations involving top radars produced the worse results. The top radar had a distant placement, whereas it covered largest RoI of human body, resulting in the highest electric field attenuation (energy loss). This produced a poor signal-to-noise ratio (SNR) that affected the prediction accuracy. In addition, we distributed the radio-frequency data into three channels to imitate RGB images, where the noise from the top radar might intervene with the latent feature mining from other radars. A low SNR could worsen the prediction, both in the single radar and dual-radar configurations. Enhancing the signal-to-noise ratio for the radar could be one method to improve classification, which could be achieved by methods such as high order cumulants (HOCs), ensemble empirical mode decomposition (EEMD), complex signal demodulation (CSD), and the state space method (SSM) [58]. Among all these methods, Liang, et al. [58] suggested that CSD worked better for IR-UWB usage. We could further enhance the radar signal with the complex signal demodulation (CSD) algorithm.

Non-lateral (prone and supine) postures aggravate OSA, while lateral (left or right) postures do not induce a negative impact on OSA. In our study, we presented a subgroup analysis graph over the lateral and non-lateral predictions. The best model achieved 213 (or 0.888 in percentage) correct prediction counts. We believe that this system could utilize this information to alert OSA patients to maintain lateral postures to mitigate the problem.

The accuracy of our study and related recent studies was compared, as shown in Table 2. The accuracy of the existing systems ranged from 0.737 [29] to 0.984 [30], while our system had an accuracy of 0.808 using the state-of-the-art vision transformer model. Studies inputting handcrafted features on machine learning models (i.e., no deep learning) demonstrated better results. The sample size might affect the model performance [59], and the size of the existing studies varied from 8 to 120, with different numbers of classified postures (from 3 to 12). In addition, a covering blanket or quilt would introduce challenges to the model predictions.

There were some limitations in our study. Our proposal aimed to identify the best radar configurations that could collect the most representative latent information on sleep posture for prediction. Nonetheless, the signals collected for each radar were collapsed into a one dimensional time-series. In order words, we could not extract the complete spatial information of the body topology. The full geometrical information of the body posture would not only improve the accuracy of sleep posture prediction but also provide explainable information for the prediction. Future improvements may consider the applications of synthetic aperture radar techniques to obtain complete spatial information through scanning and sweeping the RoI periodically [61].

In addition, a large dataset is imperative for training machine learning models, especially deep learning models [62]. In this study, we recruited 30 participants with 10 repetitions of each posture and applied data augmentation techniques. More participants with different ages, sexes, and body builds could enhance the robustness and generalizability of the prediction. Age and sex might affect the preference of sleep posture and position, but they might not confound our model since we believe that they are not associated with the body and limb position of a posture. Nevertheless, body build (or body mass index) might have an impact on our model, since it attenuates the effective area for the radar signal reflection. On the other hand, model training and prediction with fine-grained posture classes on upper limb placements could be conducted. Some participants that put their hands on the front of their chest might cause interference with the measurement of the vital sign signals by the radar, since the vital signs and the source of the vital signs are important inputs for posture estimation. Our system did not isolate the vital sign signal. Therefore, we do not know whether the ViT used the vital sign as a salient feature.

The dataset size influences the accuracy in transfer learning with deep learning models [62]. Compared to previous studies, we had a larger dataset size in addition to applying augmentation techniques, which improved the generalization of the deep learning model; however, a larger dataset size remains preferable. During the experiment, we repeated the postures 10 times but allowed freedom of limb placement, which facilitated the model generalization in terms of the postures. Some participants might place their limbs on the face or chest, which could weaken the signals from the vital signs.

The long-term objective of this research is to develop a comprehensive sleep surveillance system that can monitor sleep postures and behaviors. Our previous studies developed a depth camera system to monitor bed-exiting events [63,64] and to classify sleep postures in a field setting [14,15,28]. In the future, we will explore synthetic aperture radar and advanced modeling techniques, for instance, DensePose, which could estimate and map the human pixels of an RGB image to the 3D surface of the human body in real time [65,66].

## 5. Conclusions

Our study showed that the dual-radar configuration (side + head) with the Swin Transformer model could achieve the best sleep posture prediction accuracy of 0.808. Nevertheless, the limitations of this study included the limited data for model training in addition to the incomplete spatial information generated by the radar system. Future studies may consider a larger dataset and the application of synthetic aperture radar techniques.

## Figures and Tables

**Table 1:** Configurations of the IR-UWB radar devices.

Parameters	Top Radar	Side Radar	Head Radar
Detection Range	1.3 m–1.8 m	0.6 m–1.1 m	0.4 m–1.2 m
Transmission Power	6.3 dBm	6.3 dBm	6.3 dBm
Pulse Repetition Frequency	15.188 MHz	15.188 MHz	15.188 MHz
Bin Resolution	78 bins	78 bins	125 bins
Frame Rate	20 frames/second	20 frames/second	20 frames/second

**Table 2:** Comparison of the sleep posture accuracy between this study and other related recent studies.

Source	*n*	*np*	Stationary or Transitional	Hardware	Classifier	DL	Blanket	Accuracy
This study	30	4	Stationary	IR-UWB (Xethru X4M03)	Swin Transformer	Y	Y	0.808
Piriyajitakonkij et al. [29]	38	4	Transitional	IR-UWB (Xethru X4M03)	SleepPoseNet (Deep CNN with Multi-View Learning)	Y	N	0.737
Lai et al. [28]	18	4	Stationary	IR-UWB (Xethru X4M03)	Random Forest	N	N	0.938
Kiriazi et al. [30]	20	3	Stationary	Dual frequency Doppler radar system	Decision Tree	N	N	0.984
Zhou et al. [31]	8	8	Transitional	FMCW radar system	CNN with Inception Residual module	Y	N	0.872
Tam et al. [14]	120	7	Stationary	Depth camera (Realsense D435i)	ECA-Net50	Y	Y	0.915
Mohammadi et al. [60]	12	12	Stationary	Depth camera (Microsoft Kinect)	CNN	Y	Y	0.760

CNN: convolutional neural network; DL: deep learning; FMCW: frequency-modulated continuous-wave; IR-UWB: impulse-radio ultra-wideband; *n*: sample size; *np*: number of postures; N: No; No.: number; Y: Yes.

## Data Availability

The program, model codes, and updates presented in this study are openly available from GitHub at https://github.com/BME-AI-Lab/Vision-Transformers-for-Blanket-Penetrating-Sleep-Posture-Recognition (accessed on 1 February 2023). The video/image dataset is not publicly available since it would disclose identity and violate confidentiality.
